# 

**Published:** 1893-02

**Authors:** 


### CONTENTS FOR FEBRUARY, 1893.

ORIGINAL COMMUNICATIONS.

PAGE.

Extraction of Steel from the Interior of the Eye with the Electro-Magnet. By ALVIN A. HUBBELL, M. D. .... 389

Annual Address of the President before the Medical Society of the County of Erie. By WM. WARREN POTTER, M. D. .... 401

Clinical Remarks on Cases of Congenital Syphilis and Rickets. By A. CLIFFORD MERCER, M. D. .... 411

TRANSLATION.

Syphilis and Marriage .... 417

SOCIETY PROCEEDINGS.

The Medical Society of the County of Erie .... 424

CORRESPONDENCE.

Roswell Park, A. M., M. D.—Ferd. C. Valentine .... 431

EDITORIAL.

The Medical Society of the County of Erie .... 434

Topics of the Month .... 435

PERSONAL.

Dr. D. B. St. John Roosa .... 439

Dr. G. Frank Lydston .... 440

SOCIETY MEETINGS.

Medical Society of the State of New York .... 440

BOOK REVIEWS.

A Treatise on Diseases of the Rectum, Anus, and Sigmoid Flexure .... 442

Text-Book of Ophthalmology .... 445

Hydrotherapy of Saratoga .... 447

International Clinics .... 448

Geographical Pathology .... 450

A Pocket Medical Dictionary .... 451

Books Received .... 451

### Index to Advertisements.

PAGE.

PAGE.

